# WHY IS PSYCHOLOGICAL WELL-BEING ASSOCIATED WITH THE COMPOSITION OF OLDER ADULT’S CARE NETWORK?

**DOI:** 10.1093/geroni/igae098.0121

**Published:** 2024-12-31

**Authors:** Joukje Swinkels, Jens Abbing, Marjolein Broese Van Groenou

**Affiliations:** VU Amsterdam, Amsterdam, Noord-Holland, Netherlands; Vrije Universiteit Amsterdam, Amsterdam, Noord-Holland, Netherlands; Vrije Universiteit Amsterdam, Amsterdam, Noord-Holland, Netherlands

## Abstract

Objectives Older care recipients differ in types of care used: some receive mostly partner or other types of informal care, whereas others use mostly formal or privately paid care. This is mainly due to variation in health impairment, but it may also add to differences in psychological wellbeing. Applying Self-Determination Theory (SDT) to the care context, we hypothesize that care network types differ to the extent that they foster feelings of relatedness, autonomy and competence, resulting in different levels of psychological well-being. Methods Data are from ten three-yearly waves of the Longitudinal Aging Study Amsterdam (N= 18,434 observations from 4,837 older Dutch adults, from 1992 until 2022). Five care network types are created a priori composed of no care, (mainly) partner, informal, formal or privately paid care. Mixed-model regression analysis of depressive symptoms as measure of well-being is applied on care network type and loneliness (indicator of relatedness), mastery (autonomy) and care sufficiency (competence). Hybrid models disentangle between and within subjects effects. Results Receiving no care or care from a partner care network contributes the most to psychological wellbeing. Those with a formal care network type are worse off. Loneliness and care sufficiency partly explain these differences. Results of between and within effects are comparable. Discussion The findings support that applying SDT concepts of basic needs helps to understand how care impact psychological wellbeing. Results imply that decreasing loneliness by supporting informal care and increasing care sufficiency by improving the quality of formal care facilitates the functioning of care networks.

